# Electrospun Nanostructured Membrane Engineering Using Reverse Osmosis Recycled Modules: Membrane Distillation Application

**DOI:** 10.3390/nano11061601

**Published:** 2021-06-18

**Authors:** Jorge Contreras-Martínez, Carmen García-Payo, Mohamed Khayet

**Affiliations:** 1 Department of Structure of Matter, Thermal Physics and Electronics, Faculty of Physics, University Complutense of Madrid, Avda. Complutense s/n, 28040 Madrid, Spain; jcontr01@ucm.es (J.C.-M.); mcgpayo@fis.ucm.es (C.G.-P.); 2 Madrid Institute for Advanced Studies of Water (IMDEA Water Institute), Avda. Punto Com n° 2, Alcalá de Henares, 28805 Madrid, Spain

**Keywords:** membrane recycling, reverse osmosis, electrospinning, membrane distillation, desalination, nanofiber

## Abstract

As a consequence of the increase in reverse osmosis (RO) desalination plants, the number of discarded RO modules for 2020 was estimated to be 14.8 million annually. Currently, these discarded modules are disposed of in nearby landfills generating high volumes of waste. In order to extend their useful life, in this research study, we propose recycling and reusing the internal components of the discarded RO modules, membranes and spacers, in membrane engineering for membrane distillation (MD) technology. After passive cleaning with a sodium hypochlorite aqueous solution, these recycled components were reused as support for polyvinylidene fluoride nanofibrous membranes prepared by electrospinning technique. The prepared membranes were characterized by different techniques and, finally, tested in desalination of high saline solutions (brines) by direct contact membrane distillation (DCMD). The effect of the electrospinning time, which is the same as the thickness of the nanofibrous layer, was studied in order to optimize the permeate flux together with the salt rejection factor and to obtain robust membranes with stable DCMD desalination performance. When the recycled RO membrane or the permeate spacer were used as supports with 60 min electrospinning time, good permeate fluxes were achieved, 43.2 and 18.1 kg m^−2^ h^−1^, respectively; with very high salt rejection factors, greater than 99.99%. These results are reasonably competitive compared to other supported and unsupported MD nanofibrous membranes. In contrast, when using the feed spacer as support, inhomogeneous structures were observed on the electrospun nanofibrous layer due to the special characteristics of this spacer resulting in low salt rejection factors and mechanical properties of the electrospun nanofibrous membrane.

## 1. Introduction

Reverse osmosis (RO) is a well-established industrial membrane technology for water treatment, especially for desalination of seawater and brackish water with a 48% increase in installed capacity between 2017 and 2020. In the past year, the installed capacity was 148.4 million m^3^ per day, while in 2017, the installed capacity was 99.8 million m^3^ per day [1,2]. Although RO technology is undergoing continuous advances, there are still many aspects for improvement and some issues yet to be solved. One of the problems inherent to this technology is its high energy consumption associated to the high hydrostatic pressure required for the separation process especially for the treatment of high osmotic saline solutions. This problem has been already studied [3] and different strategies have been adopted to reduce this energy consumption [4]. Another intrinsic problem of this technology that needs a prompt resolution is the discharge of brines (i.e., aqueous saline solutions with an overall concentration in the range 50–82 g L^−1^) [5].

The continuous growth of RO technology all around the world induces a critical and irreversible increase in the quantity of discarded membrane modules, generating a large amount of waste. The replacement rate of RO modules is 20% or 10% per year, depending on whether seawater or brackish water is used as feed, respectively [6]. This replacement is normally done when the RO modules present irreversible membrane fouling and a decline of their efficiency parameters [7]. For the desalination of 1000 m^3^ per day, an average of 100 RO modules is needed [8]. Therefore, based on the installed capacity in 2020, an approximate number of 2.2 million annual waste RO modules is possible if 15% is taken as an average of the replacement rate of RO modules per year. Normally, the discarded modules are disposed of in nearby landfills. However, in order to comply with the EU regulations regarding the hierarchy of waste management (Directive 2008/98/CE: reduce, reuse, recycle, treat and recover energy), another strategy must be adopted. Taking into consideration that RO is a technology in full development, waste reduction is not possible. Some few studies have been proposed on the reuse of discarded RO modules, but without applying any previous membrane or module cleaning [9,10,11].

In 2017, more than 66% of the modules installed in RO plants were made of thin film composite (TFC)-polyamide (PA) membranes [1]. A typical TFC PA membrane is made by a support of polyester (PET) material over which a porous polysulfone (PSf) substrate is deposited by phase inversion method. Then, an active PA layer is formed on the porous PSf layer by interfacial polymerization technique [12]. This type of RO membrane was recycled using PA oxidizing agents such as sodium hypochlorite (NaOCl) to fully or partially degrade the PA layer responsible of irreversible fouling [8,10,13,14,15,16,17,18,19,20,21,22]. By applying different exposure doses to the oxidizing agent, quantified by units of parts per million of NaOCl used in the cleaning solution multiplied by the exposure time in hours (ppm·h), discarded RO membranes have been transformed to membranes for microfiltration (MF), ultrafiltration (UF) or nanofiltration (NF) separation processes [10,13,15,16,17,18,23]. In other research studies, after exposing the RO modules to NaClO oxidizing aqueous solution, the membranes were transformed to anion or cation exchanged membranes for electrodialysis (ED) or to microsystin-degrading biofilms for membrane bioreactors by applying adequate approaches [19,20,21,22,24].

Membrane distillation (MD) is a non-isothermal separation process of emerging interest. In last decade, the interest in its development based on the number of the annual published manuscripts has grown by 702.1%. Porous and hydrophobic membranes with high porosity (i.e., void volume fraction), low thermal conductivity and high liquid entry pressure (*LEP*) are required in this process in which the temperature difference, or which is the same the vapor pressure difference, is the driving force. Different MD configurations (direct contact, DCMD; air gap, AGMD; vacuum, VMD; and sweeping gas, SGMD) were considered to establish this necessary driving force. It is applied in different fields, such as in food industry and desalination. [25]. Recent studies show the interest in the development of new nanofibrous membranes derived from ecological resources or using ion exchange polymers for the treatment of industrial and textile wastewaters as an increasingly essential research area [26,27]. For instance, treatment of RO brines by MD technology is one of the feasible solutions, enabling zero liquid discharge to the environment [28]. The combination of RO and MD technologies is a solution to RO brines disposal as it can increase water recovery [29], decrease the specific energy consumption (i.e., necessary energy per volume of produced water) [30] and protect the environment. Therefore, being able to recycle and reuse parts of discarded RO modules in MD membrane engineering for the treatment of RO brines is doubly beneficial for the environment, favoring the awaited circular economy.

The objective of the present study is to apply parts of discarded RO modules, membrane and spacers, as hydrophilic supports to MD membranes. In fact, dual-layered hydrophobic/hydrophilic membranes exhibit various advantages for the MD separation process, including their high permeate flux associated with the thin hydrophobic layer and low mass transfer resistance of the hydrophilic support, their high thermal efficiency associated to the low heat transfer by conduction through the thick hydrophilic support, and their high mechanical properties. These were proved in previous research reports [31,32]. In this study, the internal components of the discarded RO modules, membranes and spacers, were cleaned and recycled as supports for the preparation MD membranes. The necessary hydrophobic layer for the MD membranes was prepared by electrospinning polyvinylidene fluoride (PVDF) nanofibrous layer over each recycled support, membrane, permeate and feed spacers. The electrospinning technique is commonly used for the preparation of nanostructured polymeric materials for different applications, including MD membrane engineering [31,32,33,34,35]. The prepared membranes were characterized by different techniques and tested in desalination of high saline solutions, brines, by DCMD.

## 2. Materials and Methods

### 2.1. End-of-Life RO Membrane Module and Hydrophilic Supports for Nanostructured MD Membranes

The discarded RO membrane modules used in a real plant located in Almeria (Spain) for brackish water treatment (spiral wound TFC-PA, 8″ diameter, TM720-400, Toray Industries, Inc., Tokyo, Japan) were cleaned and their internal parts (i.e., membrane, permeate and feed spacers) were used as supports for MD membrane preparation.

**Figure 1:**
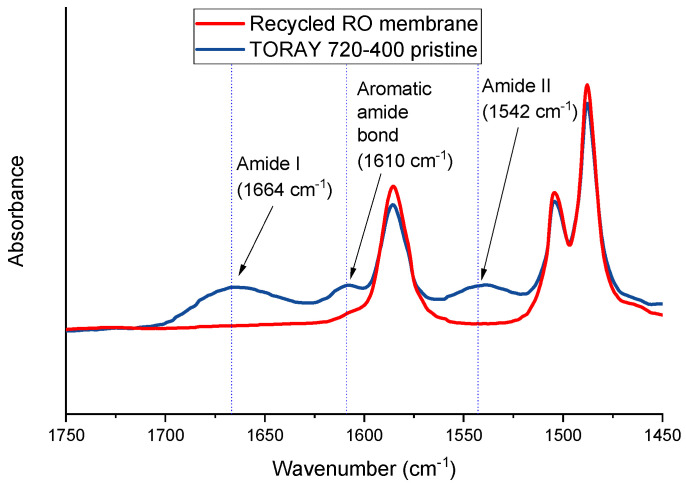
ATR-FTIR spectra of the recycled RO and TM720-400 pristine membranes.

The applied cleaning procedure was as follows. At the end of their useful life, the discarded RO modules were preserved in sodium bisulphite (500–1000 ppm). After their autopsy, these modules were washed out and stored in Milli-Q water. The used discarded RO module in this study has a water permeability of 2.04 ± 0.06 L m^−2^ h^−1^ bar^−1^, a salt rejection factor of 98.31 ± 0.24%, a colloidal fouling composition of 84% inorganic compounds and 16% organic compounds on the active layer of the RO membrane having a water contact angle of 46 ± 2° [15,16,36]. The initial water production rate of this type of membrane modules indicated by the manufacturer is 38.6 m^3^/day with a salt rejection factor of 99.7% for 37 m^2^ membrane area and 2 g L^−1^ salt (NaCl) aqueous feed solution subjected to 1.55 MPa. The discarded RO modules were then disassembled and all their internal parts were subjected to a passive cleaning procedure by submerging them in a sodium hypochlorite solution (6200 ppm of free chlorine) for 48 h (i.e., a total exposure dose of 300,000 ppm·h) in order to ensure the total oxidation of the PA layer of the RO membrane [15]. In order to confirm the total degradation of PA layer in the active layer of the recycled RO membrane, Attenuated Total Reflectance Fourier Transform Infrared Spectroscopy (ATR-FTIR) analysis was carried out for both the pristine and the recycled RO membrane. A Nicolet iS50 device (ThermoFisher Scientific Inc., Waltham, MA, USA) equipped with the deuterated triglycine sulfate–potassium bromide (DTGS–KBr) detector and a KBr beam splitter was used and the obtained spectra are shown in Figure 1.

The peaks corresponding to the PA (amide I and amide II bands and the C=C stretching vibrations of the aromatic amide bonds at 1664, 1542 and 1610 cm^−1^, respectively [17]) were clearly identified in the spectra of the pristine TM720-400 commercial RO membrane but were not detected in the spectra of the recycled RO membrane confirming the total elimination of the PA layer.

**Figure 2:**
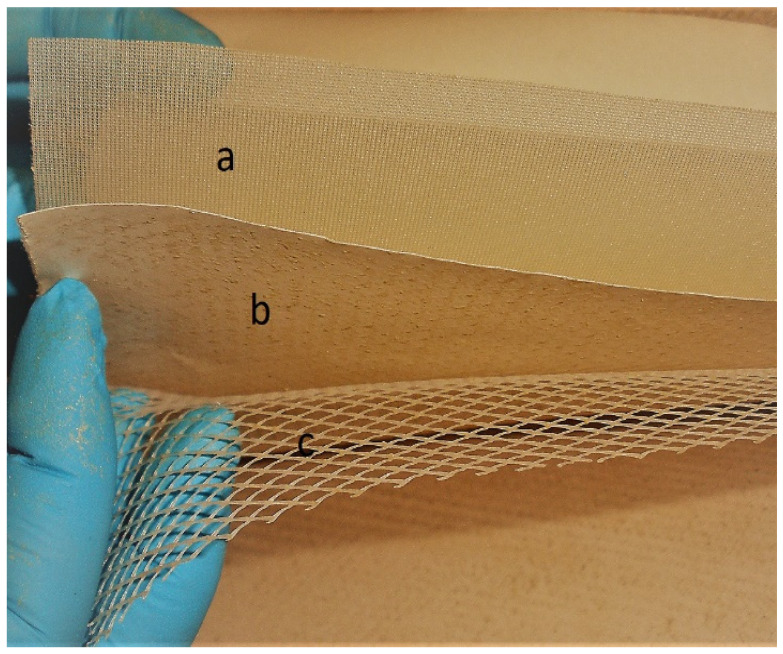
Internal parts of the discarded RO module reused as supports for the nanofibrous MD membranes: (a) permeate spacer, (b) RO membrane and (c) feed spacer.

In this study, the permeate PET spacer (a in Figure 2), the TFC membrane with degraded PA layer (b in Figure 1) and the feed polypropylene (PP) spacer (c in Figure 2) were reused [37].

### 2.2. Preparation of Nanofibrous Supported Hydrophobic/Hydrophilic MD Membranes

For the preparation of nanofibrous MD membranes, a polymer solution was prepared by dissolving 25 wt% PVDF (*M_w_* = 275 kg mol^−1^ and *M_n_* = 107 kg mol^−1^) in a solvent mixture containing 20 wt% acetone and 80 wt% N,N-dimethyl acetamide, following the optimal procedure reported by Essalhi and Khayet [38]. All chemicals were purchased from Sigma-Aldrich. The polymer solution was characterized in terms of its viscosity, electrical conductivity and surface tension at room temperature (293 K) using a viscosimeter (Brookfield, DV-I+, Brookfield Ametek Co., Middleboro, MA, USA), an electrical conductivity meter (Cyber Scan con11 Conductivity/TDS/°C, Eutech Instruments, Vernon Hills, IL, USA), and CAM 100 (a contact angle system with CAM 200 Software, KSV Instruments Ltd., Monroe, CT, USA), respectively [38]. The obtained electrical conductivity, viscosity and surface tension of the polymer solution were 9.53 ± 0.03 μS cm^−1^, 3.03 ± 0.01 Pa·s and 33.6 ± 0.6 mN m^−1^, respectively. These data are similar to those reported by Essalhi and Khayet [38] for the same polymer solution (9.57 ± 0.03 μS cm^−1^, 3.108 ± 0.005 Pa·s and 32.0 ± 1 mN m^−1^ for the electrical conductivity, viscosity and surface tension, respectively).

**Figure 3:**
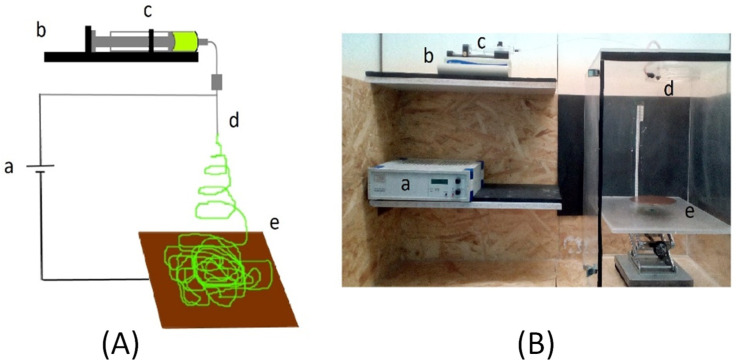
Schema (**A**) and photographs (**B**) of the electrospinning set-up (high electric voltage source (a), circulation pump (b), syringe (c), stainless steel needle (d) and cooper collector (e)).

The applied electrospinning conditions were the optimal parameters claimed by Essalhi et al. [39]. An electric pump (Fisherbrand™ Single Syringe Pump, Fisher Scientific Co., Pittsburgh, PA, USA) was used to establish a flow rate of the polymer solution of 1.23 mL h^−1^ throughout a stainless-steel needle of 0.6 mm inner diameter. The electric voltage was fixed at 24.1 kV (Iseg; model T1CP 300 304P; Iseg Spezialelektronik GmbH, Radeberg, Germany), and the air gap distance between the needle tip and the copper collector was 27.7 cm. Figure 3 shows the used electrospinning set-up for the preparation of the hydrophobic nanofibrous layer over the selected recycled RO hydrophilic supports. When using the recycled RO membrane as support, the PVDF nanofibers were deposited on the PET layer rather than on the PSf layer because of the risk to dissolve PSf layer during nanofibers deposition. The electrospinning time was varied from 30 to 90 min, and all prepared membranes were finally subjected to a thermal post-treatment at 333 K for 10 min in order to remove any residual solvents and improve the compact structure of the nanofibrous hydrophobic layer.

The electrospun nanofibrous membranes were named hereafter, RMEM, FSEM and PSEM when using the recycled RO membrane, feed spacer and permeate spacer, respectively, in which n refers to the electrospinning time 30, 60 or 90 min. For sake of comparison, unsupported hydrophobic nanofibrous membrane, NSEM, was also prepared under the same electrospinning conditions. For example, PSEM60 designates the MD membrane using the recycled permeate spacer as support and an electrospinning time of 60 min. 

### 2.3. Membrane Characterization

The water contact angle was measured at 293 K with a CAM 100 Contact Angle System that captures water drop image, which is then analyzed by CAM 200 Software (KSV Instruments Ltd., Monroe, CT, USA). Twenty-five readings were considered for each membrane, and the average water contact angle together with its standard deviation are reported.

The thickness of the recycled supports (i.e., hydrophilic layer) and the total thickness of the electrospun nanofibrous membranes (i.e., hydrophobic/hydrophilic layers) were measured using a digital micrometer (model 1724-502 series, Helios-Preisser Instruments, Gammertingen, Germany). For each sample, thirty measurements were performed in different points of each sample, and the average thickness together with its standard deviation are reported.

To determine the mean size of the inter-fiber space, the porometer POROLUX™ 100 (Porometer, Eke, Belgium) was used following the protocol detailed elsewhere [38]. Three samples of each membrane were employed and the average value with its standard deviation were calculated.

The membrane porosity or void volume fraction was determined by measuring the density of the membrane using pycnometer witch isopropyl alcohol (IPA) that penetrates the void volume of the membrane and distilled water that does not. The followed procedure was detailed in our previous study [40]. Three samples of each membrane were considered to calculate the average void volume fraction together with its standard deviation.

The *LEP_w_* of the MD membrane is the minimum pressure applied over distilled water before it penetrates into its pores having maximum size. The used set-up and the followed procedure were described in details in our previous study [41]. In this study, an initial pressure (0.01 × 10^5^ Pa) was applied for 30 min then, the pressure was increased in steps of 0.02 × 10^5^ Pa for an interval of 10 min until water drops appear at the permeate side of the membrane. For each membrane, three samples were used, and the average value with its standard deviation were calculated.

The digital microscope (VHX Digital Microscope, Keyence VH-Z100R, Itasca, IL, USA) and the field emission scanning electron microscope (FESEM, JEOL Model JSM-6335F, Jeol Ltd., Tokyo, Japan) were used in to study the surface of the samples. For SEM analysis, the membrane sample was coated with a thin gold layer using a sputter-coater (EMITECH K550 X, Emitech Groupe, Montigny, France) for 1 min under 25 mA. The nanofiber diameter was measured by the image analysis software (tpsDIG2 version 2.32, State University of New York, Stony Brook, NY, USA). In this case, two different SEM images were used for each sample, and for each image more than 100 measurements were carried out to determine the average fiber diameter and its standard deviation as described elsewhere [42]. The ENMs cross section images were obtained by previously fracturing the samples in liquid nitrogen and, later, gold coated as reported elsewhere [43].

The Instron dynamometer (model 3366, Instron Ltd., Norwood, MA, USA) was used to study the mechanical properties of the prepared membranes according to ASTMA D 3379-75 specifications at 295 K.

Direct contact membrane distillation (DCMD) configuration was considered in this study to test both the unsupported and the supported electrospun nanofibrous membranes prepared in this study using recycled supports. For comparison, the same DCMD experiments were carried out for the commercial membrane TF200 (PALL Gelman Corp., New York, NY, USA). This membrane has been widely characterized in our previous studies [39] and has 165 µm thickness, 0.23 µm mean pore size, 2.8 bar *LEP_w_* and 69% porosity. The used DCMD set-up was described in details elsewhere [32,44]. It has an effective membrane area of 1.59 × 10^−3^ m^2^. The stirring rate was 1000 rpm, the feed temperature was 353 K and the permeate temperature was 293 K. Distilled water and NaCl aqueous solutions with different concentrations varying from 6 to 150 g L^−1^ were used as feed, while distilled water was introduced in the permeate side of the membrane. The feed and permeate temperatures were controlled by two thermostats connected to the double-wall jacketed feed and permeate chambers of the DCMD set-up, respectively. The weight of the produced permeate (Δ*m*) for a predetermined time (Δ*t*) was measured using a precision balance (Sl-2002, Denver Instrument Company, Arvada, CO, USA), and the permeate flux (*J_w_*) was determined as:

(1) $$J_{w}=\frac{\Delta m}{A_{m}\Delta t}$$

where *A_m_* is the effective membrane area.

The above procedure was repeated three times for each membrane, and the average *J_w_* with its standard deviation was calculated. To determine the salt concentration in both the feed and permeate chambers, the electrical conductivity (Metrohm 712 Ω, Herisau, Switzerland) was measured before and after each permeate flux measurement and the salt rejection factor was calculated using the expression:

(2) $$\alpha =\left(1-\frac{C_{p}}{C_{f}}\right)*100$$

where *C_p_* is the concentration of the permeate solution and *C_f_* is the concentration of the feed solution. 

### 2.4. Long-Term DCMD Desalination Tests

**Figure 4:**
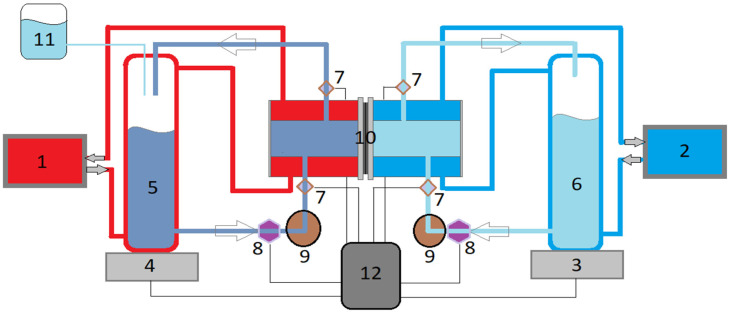
Schema of the long-term DCMD experimental system: 1. thermostat connected to the feed container; 2. cryostat connected to the permeate container; 3. precision balance; 4. precision balance; 5. feed container; 6. permeate container; 7. temperature sensors; 8. flow rate sensors; 9. double head peristatic pump; 10. membrane module; 11. reservoir; 12. computer.

Long-term DCMD desalination tests of the prepared membranes showing adequate performance were performed using the system schematized in Figure 4. The initial concentration of the feed aqueous solution was 65 g L^−1^ while the permeate was distilled water. This initial NaCl feed concentration was selected as it is an average concentration of RO brines of seawater desalination plants (50–82 g L^−1^ [5]). The membrane with an effective area of 1.26 × 10^−3^ m^2^ was placed between the double wall jacketed feed and permeate chambers. The feed temperature was maintained at 353 K while that of the permeate was 293 K by means of a thermostat (TE-8D, Techne Inc., Burlington, NJ, USA) and a cryostat (N0691883 Recirculator, Poly Science^®^, Niles, IL, USA), respectively. The temperatures were measured by two pt100 sensors placed inside the chambers of the membrane module. A double head peristaltic pump (model 323S, Watson-Marlow Fluid Technology Group, Wilmington, DE, USA) was used for the circulation of both the feed and permeate solutions with a flow rate of 0.8 L min^−1^. The collected permeate each minute was measured by a precision balance (Sartorius Lab Instruments GmbH & Co., Goettingen, Germany, respectively) connected to a computer. The electrical conductivity of both the feed and permeate solutions was measured every hour (Metrohm 712 Ω, Herisau, Switzerland). The permeate flux and the salt rejection factor were also determined using Equations (1) and (2), respectively. The data were recorded every minute and then the mass differences and their associated permeate fluxes were determined. From these data, the average *J_w_* was calculated each hour with its standard deviation.

Two types of long-term DCMD tests, regeneration test and exhaustion test, were carried out. For the first test, the feed salt concentration was kept constant around 65 g L^−1^ by introducing an adequate amount of distilled water in the feed tank comparable to that of the permeate; the second test consists of the concentration of the feed solution as much as possible until the whole feed solution was recovered, except the dead-end feed volume (around 300 mL and over 160 g L^−1^ concentration). In both DCMD tests, the initial feed volume was 2 L. In this test, a linear behavior was considered to quantify the decline of the permeate flux with time, and the corresponding absolute value of the slope was taken as the decay slope of the permeate flux (*DS_Jw_*). The exhaustion operating time and the final feed concentration correspond to a substantial decline of the permeate flux with time (i.e., a reduction of the slope presenting the permeate flux with time by around 10%). These two tests are complementary and permit us to know the permeate fluxes at different salt concentrations together with the maximum feed concentration that each membrane can reach, as well their DCMD performance and long-term stability.

## 3. Results and Discussion

### 3.1. Membrane Characterization

**Figure 5:**
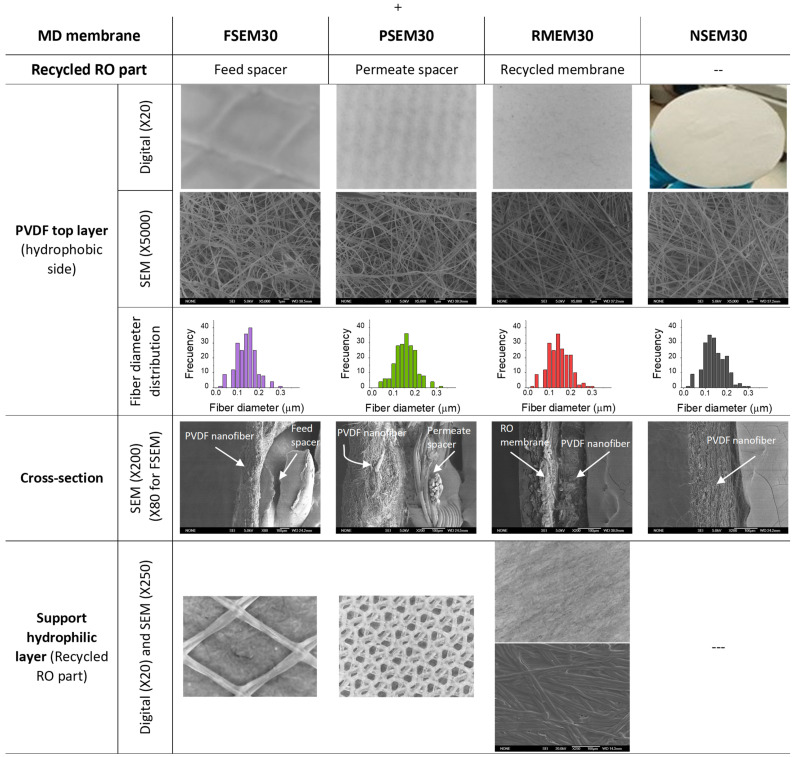
Digital and SEM images of the top surface of the unsupported membrane (NSEM30), feed spacer supported membrane (FSEM30), permeate spacer supported membrane (PSEM30) and RO recycled supported membrane (RMEM30) with the corresponding fiber diameter distribution of the electrospun PVDF top layer and their cross-sections SEM images and the digital images of the feed and permeate spacers and the RO membrane of the discarded RO modules reused as support of the PVDF ENMs.

The morphological structure of the recycled supports and the nanostructured membranes are shown in Figure 5, and the results of the characterization tests of the electrospun nanofibrous membranes for 30 min are summarized in Table 1.

The morphological structure of the used supports is evident from the digital microscopic images shown in Figure 5. From the FSEM30 images, a large accumulation of nanofibers could be observed on the mesh nodes of the recycled feed spacer. This agrees with the thickness measurements as will be discussed later on, and it is associated to the more open structure of this spacer. When using the permeate spacer as a support, whose mesh structure is denser and more compact, this problem was not detected, and an even and homogeneous electrospun PVDF nanostructure was formed as shown by the corresponding SEM image (PSEM30). Similar to NSEM30 and PSEM30, an even and homogeneous PVDF nanostructure was electrospun on the recycled RO membrane RMEM30. The fiber diameter distributions of the electrospun PVDF ENMS presented in Figure 5 were found to be similar and independent on the used support (i.e., the average fiber diameter was 0.15 ± 0.05, 0.16 ± 0.05, 0.15 ± 0.05 and 0.15 ± 0.05 μm, for FSEM30, PSEM30, RMEM30 and NSEM 30 membranes, respectively).

The representative cross-sectional SEM images of NSEM30 membrane and the supported membranes (FSEM30, PSEM30, RMEM30) shown in Figure 5 confirmed further the previously discussed structural characteristics of the membranes. The PVDF nanofibrous top layer exhibited a randomly oriented 3D non-woven nanofibrous structure. Regarding the junction between the PVDF nanofibrous top layer and the different hydrophilic supports used, it is clearly confirmed that the PVDF nanofibrous layer was not attached adequately to the feed spacer although the joints of the feed spacer bounded the PVDF nanofibrous layer. The best junction between the top and bottom layers was achieved for the RMEM30 using the PET side of the recycled RO membrane as contact layer for the PVDF nanofibrous in the electrospinning process.

Taking into consideration the standard deviation, the thickness of the hydrophobic PVDF layer of all electrospun nanofibrous membranes is similar. This is attributed partly to the comparable diameter of the formed PVDF nanofibers, 0.15 ± 0.05 µm, of all membranes regardless of the recycled support. The observed difference of their total thickness is associated to that of the recycled support, which varies in the order: recycled RO membrane < permeate spacer < feed spacer. Compared to the other electrospun nanofibrous membranes, the high standard deviation obtained for the total thickness of the FSEM30 membrane is associated with the open structure of the feed spacer resulting in an uneven thickness of this membrane (see Figure 5). Both the feed and permeate spacers are totally hydrophilic as the water drop cannot stand over them, while the water contact angle of the PSf side of the RO recycled support is 65°, which is much lower than that of the PVDF nanofibrous layer (132–140°). The lower water contact angle of the FSEM30 membrane (i.e., 6% lower) compared to that of the other nanofibrous membranes, which was around 140°, may be attributed to the inhomogeneous and uneven nanostructure of the electrospun PVDF layer of this membrane. This is also confirmed by its higher inter-fiber space, lower *LEP_w_* and poor mechanical properties as is discussed later on. 

From the membrane characteristics shown in Table 1, it can be seen that the used recycled support affects the nanostructure of the electrospun PVDF layer although the same electrospinning conditions were applied. Compared to the unsupported PVDF nanofibrous membrane, NSEM30, taken as reference, the inter-fiber space of the PSEM30 and FSEM30 was greater, while that of the RMEM30 was smaller due to the different structures of the recycled supports as can be seen in Figure 5. The RMEM30 membranes exhibited a very small inter-fiber space because of the low pore size and compact structure of the PSf layer of the recycled support. This also affected the void volume fraction of this membrane, which was much smaller than that of the other electrospun nanofibrous membranes. 

The void volume fraction of the NSEM30 membrane is in accordance with those reported elsewhere [24,30]. The two recycled permeate and feed spacers reduced only slightly the void volume fraction, 2.4 and 4.9%, respectively; whereas this reduction was much higher, 22.0%, when using the recycled RO membrane as support. 

Compared to other nanofibrous membranes [38,41], the *LEP_w_* value of the NSEM30 is smaller due to the lower electrospinning time and heat post-treatment applied in this study. The RMEM30 membrane exhibits the highest *LEP_w_* value because of its lowest inter-fiber space as explained previously. In contrast, the FSEM30 presents the lowest *LEP_w_* value because of its highest inter-fiber space and lowest hydrophobic character of the PVDF nanofibrous layer. Both NSEM30 and PSEM30 membranes showed almost the same *LEP_w_* value. This may be attributed to the same hydrophobic character of these two membranes although the inter-fiber space of the NSEM30 membrane is 16.9% smaller.

**Figure 6:**
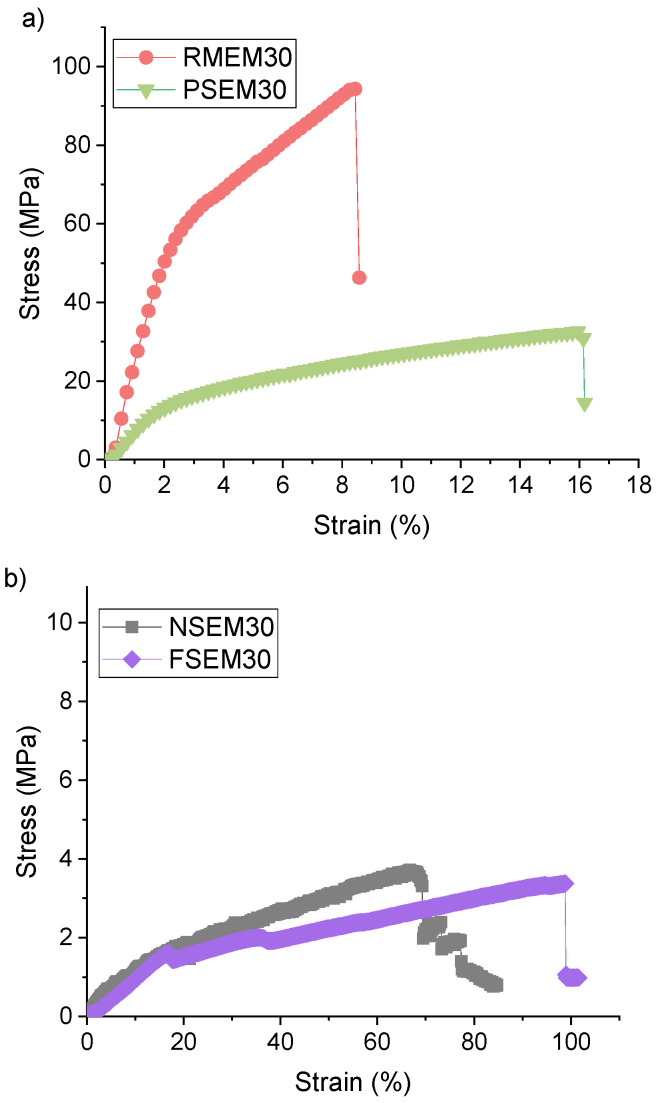
Stress–strain curves of the unsupported and supported electrospun nanofibrous membranes for 30 min electrospinning time. (**a**) RMEM and PSEM using the RO membrane and permeate spacer, respectively, with 30 min electrospinning time; (**b**) NSEM and FSEM using unsupported and feed spacer, respectively, with 30 min electrospinning time.

The mechanical properties of the prepared membranes are also summarized in Table 1, and the corresponding stress/strain curves are plotted in Figure 6. Based on the mechanical characteristics reported for other PVDF nanofibrous membranes [41], NSEM30 showed the expected values for Young’s modulus (*Y_M_*) and tensile strength (*Ts*),17 ± 1 MPa and 4 ± 1 MPa, respectively. This membrane was prepared with 30 min electrospinning time, and it is known that the mechanical properties of electrospun nanofibrous membranes are improved with the increase in the electrospinning time, which enhances the membrane thickness. One of the advantages of the use of supports is to improve the membrane mechanical properties. In this case, the use of the recycled RO membrane (RMEM30) and the permeate spacer as supports (PSEM30) induced a considerable enhancement of *Y_M_* and *Ts* compared to those of the unsupported NSEM30 membrane. However, the elongation at break (*E_b_*) of these membranes was reduced. This is attributed to the stiffness and low elasticity of their corresponding recycled supports made up with PET polymer and to the morphological structure of the permeate spacer composed of a fine mesh and a high number of nodes. On the contrary, for the FSEM30, both *Y_M_* and *Ts* were decreased indicating a worse mechanical strength compared to the other membranes. This is attributed to the uneven structure of the PVDF nanofibrous structure and to the open and wide web structure of the corresponding PP support.

Table 2 shows the DCMD permeate flux and the salt rejection factor of the unsupported and supported nanostructured membranes prepared with 30 min electrospinning time for different NaCl feed concentrations. For sake of comparison, the corresponding permeate flux of the commercial membrane TF200 is also presented. It can be seen that the PSEM30 and NSEM30 membranes exhibit the highest permeate fluxes even higher than those of the commercial membrane TF200 (i.e., over 28%). However, the salt rejection factor of these NSEM30 and PSEM30 membranes were lower than those of TF200. This is due partly to the low thickness of the PVDF nanofibrous layer and low *LEP_w_* values inducing wetting of large size inter-fiber space of the PVDF layer. Compared to TF200, the FSEM30 membrane has almost similar permeate fluxes, but much lower salt rejection factors, even lower than those of the NSEM30 and PSEM30 membranes. This is due to its lower *LEP_w_* and its uneven and inhomogeneous PVDF nanostructured layer. The permeate flux of the RMEM30 membrane was the lowest but it exhibited high salt rejection factors due to its smallest inter-fiber space and void volume fraction compared to the other membranes. As it is well known, the decrease in the permeate flux with the increase in the NaCl concentration of the feed solution from 0 to 150 g L^−1^ is due to the reduction of the vapor pressure of the feed solution and the concentration polarization effect resulting in a decline of the driving force for DCMD. The observed different reduction rates of the permeate flux with the increase in the feed salt concentration (40% for RMEM30, 25% for NSEM30, 26% for PSEM30, 30% for FSEM30 and 22% for TF200) may be attributed to the different supports used and to the membrane characteristics affecting the temperature and concentration polarization effects.

### 3.2. Long-Term DCMD Desalination Tests

Based on the characterization results presented in the previous section, the FSEM30 membrane prepared with the recycled feed spacer as a support was discarded for further long-term DCMD desalination tests. As is mentioned in the previous section, this is because of the uneven and inhomogeneous nanostructure of its electrospun PVDF layer, low *LEP_w_*, bad mechanical properties, large inter-fiber space and overall low salt rejection factors. 

The PVDF nanostructured layer thickness of the other three membranes (NSEM, RMEM, PSEM) was increased by prolonging the electrospinning time to 60 and 90 min. These membranes were then characterized and tested for long-term DCMD desalination tests, as explained previously. The obtained results are shown in Table 3. As was expected, the membranes prepared with larger electrospinning time were thicker. The measured water contact angles of the top (137–140°) and bottom layers of the prepared membranes were the same as those of the membranes prepared with 30 min electrospinning time (Table 1). Both the inter-fiber space and *LEP_w_* vary with the electrospun thickness independently of the recycled support used or whether the membrane is supported or not. The inter-fiber space decreased, whereas the *LEP_w_* increased with the increase in the electrospinning time from 30 to 90 min. These results were already observed by Essalhi and Khayet for the unsupported PVDF nanofibrous membranes prepared with electrospinning time varying from 1 to 4 h [41,45]. In addition to the significant increase in the *LEP_w_* with electrospinning time (i.e., 300% for NSEM and PSEM membranes and 24.6% for RSEM membranes when the electrospinning time was increased from 30 to 60 min), the *LEP_w_* values of the supported nanofibrous membranes, RMEM and PSEM, are greater than those of the self-sustained NSEM membranes. These results are attributed to the used recycled support and to the reduction of the maximum inter-fiber space with electrospinning time (i.e., increase in the PVDF nanostructured layer) provided that the hydrophobic character of the PVDF layer is maintained almost the same (i.e., the water contact angle was maintained around 138°). The highest *LEP_w_* values were obtained for RMEM membranes because of the PSf layer of the corresponding recycled support that induced smaller inter-fiber space of the electrospun PVDF layer. However, no clear trend was detected for the void volume fraction with electrospinning time. This may be attributed to the short electrospinning time variation in this study (30–90 min) and the effect of the recycled supports. Essalhi and Khayet [41,45] reported a gradual and slight enhancement of the void volume fraction with the increase in the electrospinning time or which is the same the increase in the thickness of unsupported PVDF nanofibrous membranes (i.e., an enhancement of 9% when the electrospinning time was increased from 1 h to 4 h). This was attributed to the fact that the continuous deposited nanofibrous layer on the metallic collector acted as an electric insulator towards the dissipation of the electric charges to the collector resulting in a less packed nanofiber network. For a thicker nanofibrous layer or support, more electrostatic charges accumulated in the formed nanofibers causing them to repel each other so that a more loosely packed nanofibrous structure was induced resulting in a higher void volume fraction.

**Figure 7:**
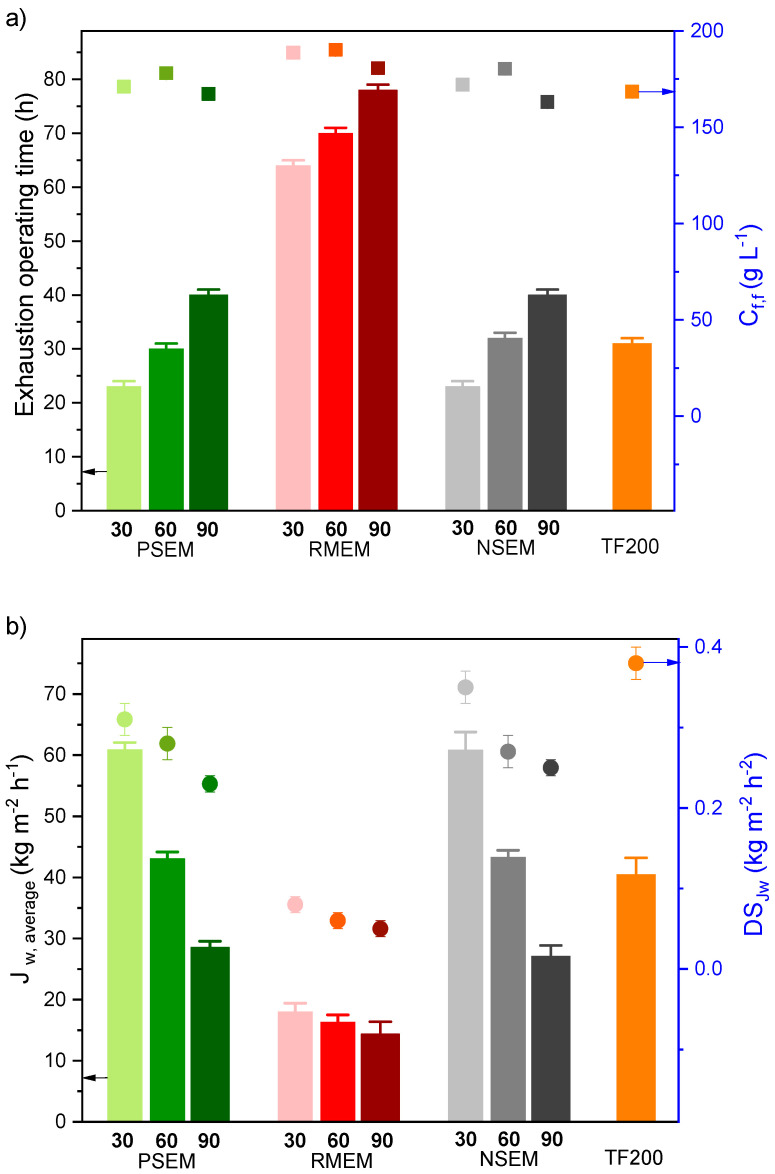
Long-term DCMD exhaustion test of the unsupported, NSEM, the supported electrospun nanofibrous membranes, PSEM and RMEM, and the commercial membrane TF220: (**a**) operating time (vertical bar) and final feed salt concentration (*C_f,f_*, symbols) (**b**) average permeate flux (*J_w, average_*, vertical bars) and decay slope of the permeate flux (*DS_Jw_*, symbols). Experimental conditions: *T_f_* = 80 °C, *T_p_* = 20 °C, *A_m_* = 1.26 × 10^−3^ m^2^, feed and permeate flow rate fixed at 0.8 L min^−1^, distilled water as permeate and initial *C_f_* at 65 g L^−1^.

The results of the long-term DCMD exhaustion test of the NSEM, RMEM, PSEM and TF200 are plotted in Figure 7. As stated previously, this test consists of the concentration of the feed solution from 65 g L^−1^ up to a maximum concentration over 160 g L^−1^. In order to compare all the membranes, the exhaustion operating time and the final feed salt concentration (*C_f,f_*) were considered when the slope of the permeate flux changed significantly (around 10%). For all membranes, these results showed how the DCMD operating time increases, and the average permeate flux decreases with increasing the electrospinning time of the PVDF nanostructured layer. This was expected since the increase in the PVDF layer thickness, reduces the permeate flux and prolong the necessary time for the concentration of the feed solution over 160 g L^−1^. When the recycled membrane was used as a support, the obtained permeate flux for the RMEM membranes was the lowest compared to the other membranes. The highest final feed salt concentrations achieved for these membranes was up to 190 g L^−1^ for the membranes RMEM30 and RMEM60. When the permeate spacer was used as a support (PSEM), the final feed salt concentration and the necessary DCMD operating time were similar to those of the unsupported NSEM membranes and lower than those of the RMEM membranes. In this case, the PSEM60 and NSEM60 exhibited the same DCMD operating time to that of the membrane TF200 with almost the same average permeate flux but greater final feed salt concentrations.

For all prepared membranes, the slight decrease in the final concentration of the feed solution of the nanofibrous membranes prepared with 90 min electrospinning time, compared to those prepared with 30 and 60 min, is due partly to the corresponding lower slope of the permeate flux decay with time (*DS_Jw_*) and to the thermal post-treatment that might be insufficient to induce the necessary junction points between nanofibers of the thicker membranes prepared with 90 min electrospinning time increasing, therefore, scaling phenomenon and causing an earlier membrane collapse. The permeate fluxes obtained for all PSEM membranes are similar to NSEM membranes and those prepared with 30 and 60 min electrospinning exhibited superior permeate fluxes to that of the commercial TF200 membrane. All prepared membranes exhibited lower *DS_Jw_* values than that of the membrane TF200 being those of the RMEM membranes the lowest. This indicates the more stable DCMD performance with time and with the increase in the feed concentration of the membranes prepared with recycled supports. 

**Figure 8:**
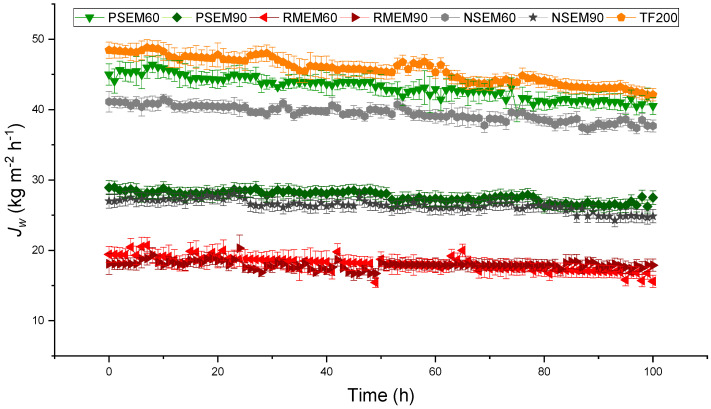
Long-term DCMD regenerative test of the unsupported (NSEM60, NSEM90), and the supported electrospun nanofibrous membranes (PSEM60, PSEM90, RMEM60, RMEM90) and the commercial membrane TF200). Experimental conditions: *T_f_* = 80 °C, *T_p_* = 20 °C, *A_m_* = 1.26 × 10^−3^ m^2^, feed and permeate flow rate fixed at 0.8 L min^−1^, *C_f_* at 65 g L^−1^ and distilled water as permeate.

For the long-term DCMD regenerative test, in which the feed salt concentration was maintained at 65 g L^−1^, only the electrospun nanofibrous membranes with 60 and 90 min electrospinning time were considered together with the commercial TF200 membrane. The prepared membranes with 30 min electrospinning time could not withstand 100 h DCMD operation because of the inter-fiber space wetting and the passage of salt to the permeate resulting in a low salt rejection factor of 90% after only 56 h DCMD operation. Figure 8 shows the permeate flux over 100 h DCMD operation for the unsupported (NSEM60, NSEM90), the supported (PSEM60, PSEM90, RMEM60, RMEM90) nanofibrous membranes and the commercial membrane TF200. The salt rejection factor of all these membranes was maintained greater than 99.99%. A strong dependence of the permeate flux with the electrospinning time was detected (i.e., decrease in the permeate flux with the electrospinning time or thickness of the PVDF nanofibrous layer). From Figure 8, three groups of membranes can be classified from higher to lower permeate fluxes: (TF200, PSEM60, NSEM60) > (PSEM90, NSEM90) > (RMEM60, RMEM90). A considerable permeate flux decline was observed for the first two groups whereas practically the same permeate flux was maintained (≈19 kg m^−2^ h^−1^) for the third group presented by the RMEM60 and RMEM90 membranes. The calculated slope of the permeate flux decay with time (*DS_Jw_*) was 0.062 ± 0.002, 0.049 ± 0.002 and 0.031 ± 0.002 kg m^−2^ h^−2^ for the TF200, PSEM60, NSEM60 membranes, 0.029 ± 0.002 and 0.023 ± 0.002 kg m^−2^ h^−2^ for the PSEM90 and NSEM90 membranes and 0.029 ± 0.002, 0.006 ± 0.001 kg m^−2^ h^−2^ for the RMEM60 and RMEM90 membranes, respectively. This decreased with the increasing of the electrospinning time, and all prepared membranes showed lower *DS_Jw_* values than the commercial TF200 membrane. Among all prepared membranes, RMEM90 exhibited the lowest permeate flux decay with time while the PSEM60 membrane presented the highest one maintaining very high and stable salt rejection factors.

Taking into consideration the achieved high salt rejection factors greater than 99.99%, the reduction of the permeate flux with time may be attributed to scaling effect, which was more pronounced for the group of membranes exhibiting high permeate fluxes since the measured electrical conductivity of the permeate throughout the experiments showed very small variations (6–14 µS cm^−1^). In addition, some inter-fiber spaces of some membranes may be wetted reducing slightly the permeate flux and maintaining the salt rejection factor higher than 99.99%.

The DCMD performance of the prepared nanostructured membranes in this study, both the unsupported (NSEM60) and the supported ones using the recycled RO membrane (RMEM60) and the permeate spacer (PSEM60), were compared in Table 4 to other PVDF and polyvinylidenefluoride-co-hexafluoropropyle (PVDF-HFP) electrospun nanofibrous MD membranes reported in other manuscripts [31,41,46,47,48,49,50,51,52,53,54]. It can be confirmed that both RMEM60 and PSEM60 exhibit DCMD permeate fluxes within the range of those reported for PVDF and PVDF-FHP nanofibrous membranes (13.3–54.4 kg m^−2^ h^−1^) with good and stable salt rejection factors.

## 4. Conclusions

In this study, we propose recycling and reusing RO membranes and spacers of discarded RO modules as supports in MD membrane engineering, extending their useful life cycle and contributing to the awaited circular economy of waste management. In addition, the proposed membranes were tested for the treatment of high saline aqueous solutions, near saturation, in order to check their ability to process RO brines, which is also beneficial for the environment favoring the noted zero liquid discharge for RO desalination plants.

The recycled RO membrane and the permeate spacer of the RO discarded module used as hydrophilic supports for the PVDF nanofibrous membranes exhibit suitable characteristics for MD separation process. The MD membranes supported by the recycled RO membrane (RMEMs) have a smaller inter-fiber space and a greater *LEP_w_* than the membrane supported by the permeate spacer (PSEMs). Compared to the unsupported PVDF membrane, the use of these two supports enhanced both Young´s modulus (*Y_M_*) and tensile strength (*Ts*). The DCMD tests showed higher salt rejection factors for the RMEMs and similar permeate fluxes to those of the unsupported PVDF membranes were observed for the PSEMs. Both supported membranes exhibited stable DCMD performance with time.

The recycled feed spacer of the RO discarded module caused a large accumulation of PVDF nanofibers on its mesh nodes due to its open structure resulting in an uneven thickness of the PVDF nanostructured membranes (FSEMs). Therefore, this type of membrane showed lower *LEP_w_* value, a greater inter-fiber space, lower *Y_M_* and *T_s_* values and a lower salt rejection factor than the other membranes.

Compared to other PVDF or PVDF-HFP electrospun nanofibrous membranes reported in the literature, the prepared membranes, RMEM60 and PSEM60, with 60 min electrospinning time presented reasonably good DCMD performance (i.e., 43.2 and 18.1 kg/m^2^ h with high salt rejection factors for RMEM60 and PSEM60, respectively).

## Figures and Tables

**Table 1:** Characteristics of electrospun unsupported and supported nanofibrous membranes for 30 min electrospinning time: Thickness (*δ*) and water contact angle (*θ_w_*) of the top and bottom surfaces of the membrane, total thickness (*δ_t_*), mean inter-fiber space (*d_i_*), void volume fraction (*ε*), liquid entry pressure of water (*LEP_w_*), Young´s modulus (*Y_M_*), tensile strength (*Ts*) and elongation at break (*E_b_*).

Membrane Code	Bottom Layer (Hydrophilic Support)		Top Layer (PVDF Hydrophobic Membrane)		*δ_t_* (μm)	*d_i_* (μm)	*ε* (%)	*LEP_w_* (10^3^ Pa)	*Y_M_* (MPa)	*Ts* (MPa)	*E_b_* (%)
	*δ_S_* (μm)	(*θ_w_*)*_S_* (°)	*δ_PVDF_* (μm)	(*θ_w_*)*_PVDF_* (°)							
NSEM30	-	-	28 ± 5	139 ± 5	28 ± 5	2.02 ± 0.01	82 ± 1	14 ± 2	17 ± 1	4 ± 1	77± 15
RMEM30	98 ± 3	65 ± 7	31 ± 7	140 ± 5	128 ± 4	<0.1 ^c^	64 ± 1	65 ± 4	3721 ± 86	93 ± 3	8 ± 1
PSEM30	221 ± 5	- ^b^	33 ± 10	140 ± 2	255 ± 4	2.43 ± 0.08	80 ± 4	15 ± 1	881 ± 42	32 ± 1	15 ± 1
FSEM30	774 ± 7 ^a^	- ^b^	32 ± 16	132 ± 5	806 ± 9 ^a^	3.58 ± 0.17	78 ± 4	7 ± 2	11 ± 1	3 ± 1	88 ± 8

^a^ Thickness measured at the nodes of the mesh. ^b^ 100% hydrophilic nets with open structures (see the corresponding images in Figure 5). ^c^ Mean size below the measurable limit of Porolux100 (100 nm).

**Table 2:** DCMD permeate flux (*J_w_*) and salt rejection factor (α) of the unsupported and supported nanostructured membranes prepared with 30 min electrospinning time together with that of the commercial membrane TF200 for different NaCl feed concentrations.

NaCl Concentration (g L^−1^)	NSEM30		RMEM30		PSEM30		FSEM30		TF200	
	*J_w_* (kg m^−2^ h^−1^)	α (%)	*J_w_* (kg m^−2^ h^−1^)	α (%)	*J_w_* (kg m^−2^ h^−1^)	α (%)	*J_w_* (kg m^−2^ h^−1^)	α (%)	*J_w_* (kg m^−2^ h^−1^)	α (%)
0	72.5 ± 0.6	-	29.2 ± 0.2	-	74.7 ± 0.8	-	63.4 ± 0.5	-	55.9 ± 0.1	-
6	69.1 ± 0.4	99.97	25.7 ± 0.3	99.98	70.0 ± 0.2	99.98	58.3 ± 0.1	99.78	52.4 ± 0.1	99.99
12	66.6 ± 0.4	99.94	24.7 ± 0.2	99.97	68.5 ± 0.2	99.93	54.9 ± 0.2	99.74	51.6 ± 0.1	99.99
30	64.8 ± 1.1	99.94	23.6 ± 0.2	99.97	66.5 ± 0.3	99.93	51.3 ± 0.2	99.78	51.0 ± 0.3	99.99
60	61.9 ± 0.2	99.95	22.1 ± 0.2	99.99	63.6 ± 0.1	99.95	49.3 ± 0.4	99.71	50.0 ± 0.1	99.99
90	59.3 ± 0.3	99.95	20.9 ± 0.2	99.99	59.7 ± 0.2	99.95	46.5 ± 0.3	99.79	48.6 ± 0.1	99.99
120	56.6 ± 0.2	99.96	19.8 ± 0.5	99.99	57.7 ± 0.1	99.95	45.8 ± 0.3	99.77	46.3 ± 0.1	99.99
150	54.8 ± 0.3	99.97	17.3 ± 0.2	99.99	55.2 ± 0.1	99.95	44.0 ± 0.3	99.66	43.8 ± 0.1	99.99

**Table 3:** Characteristics of the electrospun unsupported and supported nanofibrous membranes prepared with 60 and 90 min electrospinning time: Thickness (*δ*) and water contact angle (*θ_w_*) of the top and bottom surfaces of the membrane, total thickness (*δ_t_*), mean inter-fiber space (*d_i_*), void volume fraction (*ε*) and liquid entry pressure of water (*LEP_w_*).

Membrane Code	Bottom Layer (Hydrophilic Support)		Top Layer (PVDF Hydrophobic Membrane)		*δ_t_* (μm)	*d_i_* (μm)	*ε* (%)	*LEP_w_* (10^3^ Pa)
	*δ_S_* (μm)	(*θ_w_*)*_s_* (°)	*δ_PVDF_* (μm)	(*θ_w_*)_*PVDF*_ (°)				
NSEM60	-	-	68 ± 5	140 ± 2	68 ± 5	1.49 ± 0.28	87 ± 1	44 ± 3
NSEM90	-	-	125 ± 6	139 ± 4	125 ± 6	1.14 ± 0,35	84 ± 1	56 ± 2
RMEM60	98 ± 3	65 ± 7	74 ± 9	137 ± 2	171 ± 6	<0.1 ^b^	69 ± 2	71 ± 2
RMEM90	98 ± 3	65 ± 7	111 ± 10	139 ± 2	209 ± 7	<0.1 ^b^	65 ± 4	81 ± 3
PSEM60	221 ± 5	- ^a^	84 ± 19	137 ± 3	306 ± 14	2.14 ± 0.78	77 ± 3	49 ± 1
PSEM90	221 ± 5	- ^a^	142 ± 20	138 ± 6	363 ± 15	1.46 ± 0.23	82 ± 3	60 ± 3

^a^ 100% hydrophilic nets with open structures (see the corresponding images in Figure 4). ^b^ Mean size below the measurable limit of Porolux100 (100 nm).

**Table 4:** DCMD performance and characteristics of optimized electrospun nanofibrous membranes reported in other studies together with the best ones obtained in the present study for the supported PVDF nanofibrous membranes using the recycled RO membrane (RMEM60) and the permeate spacer (PSEM60), and the unsupported PVDF nanofibrous membrane (NSEM60).

Material	Support	Membrane Characteristics	DCMD Conditions	Permeate Flux (kg m^−2^ h^−1^)	Salt Rejection Factor (%)	Ref.
PVDF	Recycled RO permeate spacer (PSEM60)	*ε*: 77%; *δ*: 306 μm; *LEP_w_*: 0.49 bar; *θ_w_*: 137°.	*c_f_:* 65 g/L; Δ*T* = 60 K	43.2 ± 1.5	99.99	This study
PVDF	Recycled RO membrane (RMEM60)	ε: 69%; *δ*: 171 μm; *LEP_w_*: 0.71 bar; *θ_w_*: 137°.	*c_f_:* 65 g/L; Δ*T* = 60 K	18.1 ± 0.9	99.99	This study
PVDF	Unsupported (NSEM60)	*ε*: 87%; *δ*: 68 μm; *LEP_w_*: 0.44 bar; *θ_w_*: 140°.	*c_f_:* 65 g/L; Δ*T* = 60 K	39.4 ± 1.1	99.99	This study
PVDF	Unsupported	*ε*: 86%; *δ*: 144.4 μm; *LEP_w_*: 0.63 bar; *θ_w_*: 139.7°.	*c_f_:* 12 g/L; Δ*T* = 60 K	15.2	99.7	[41]
PVDF-HFP	Unsupported	*ε*: 90%; *δ*: 55 μm; *θ_w_*: 128°.	*c_f_*: 30 g/L; Δ*T* = 55 K	13.3	99.99	[46]
PVDF	PSf nanofibers	*ε*: 92%; *δ*: 407 μm; *LEP_w_*: 0.79 bar; *θ_w_*: 140.5°.	*c_f_*: 30 g/L; Δ*T* = 60 K	47.7	99.99	[31]
PVDF	Polyester mesh (big square pores)	*ε*: 88.6%; *δ*: 190 μm; *LEP_w_*: 0.45 bar; *θ_w_*: 145°.	*c_f_*: 35 g/L; Δ*T* = 60 K	49.3	99.99	[47]
PVDF/TBAC/FA	Unsupported	*ε*: 71.28%; *δ*: 60 μm; *LEP_w_*:2.1 bar; *θ_w_*: 137°.	*c_f_*: 35 g/L; Δ*T* = 40 K	54.4	99.5	[48]
PVDF/SiO_2_NPs	Unsupported	*ε*: 80%; *δ*: 129 μm; *LEP_w_*: 0.842 bar; *θ_w_*: 156.4°.	*c_f_*: 30 g/L; Δ*T* = 60 K	30.7	99.9	[49]
PVDF-HFP	Unsupported	*ε*: 58%; *δ*: 65 μm; *LEP_w_*: 1.3 bar; *θ_w_*: 120°.	*c_f_*: 35 g/L; Δ*T* = 41 K	22	98	[50]
PVDF-HFP/CNT	Unsupported	*ε*: 89%; *δ*: 83 μm; *LEP_w_*: 0.4 bar; *θ_w_*: 150.4°.	*c_f_*: 35 g/L; Δ*T* = 40 K	48.1	99.99	[51]
PVDF-HFP/AC	Nylon flat sheet	*ε*: 90%; *δ*: 200 μm; *LEP_w_*: 1.36 bar; *θ_w_*: 142.7°.	*c_f_*: 35 g/L; Δ*T* = 45 K	45.6	99.99	[52]
PVDF-HFP	PAN nanofibers	*ε*: 90%; *δ*: 82 μm; *LEP_w_*: 0.94 bar; *θ_w_*: 150°.	*c_f_*: 35 g/L; Δ*T* = 40 K	30	98.5	[53]
PVDF-HFP/SiF	Unsupported	*ε*: 71%; *δ*: 130 μm; *LEP_w_*: 2.7 bar; *θ_w_*: 100°.	*c_f_*: 35 g/L; Δ*T* = 50 K	26	99.99	[54]

PVDF-HFP: polyvinylidenefluoride-co-hexafluoropropylene; TBAC: tetrabutylammonium chloride; FA: fluorinated acrylate copolymer; SiO_2_NPs: silica nanoparticles; PS: polystyrene; SBS: styrene-butadiene-styrene; CNT: carbon nanotubes; AC: activated carbon; PAN: polyacrylonitrile; SiF: silica fibers; *ε*: porosity; *δ*: thickness; *θ_w_*: water contact angle; *LEP_w_*: liquid entry pressure; Δ*T*: temperature difference between the feed and permeate; *c_f_*: feed salt concentration.

## Data Availability

The data is available on reasonable request from the corresponding author.

## Nomenclature

Symbols:	
AC	Activated carbon
AGMD	Air gap membrane distillation
*A_m_*	Effective membrane area (m^2^)
ATR-FTIR	Attenuated Total Reflectance Fourier Transform Infrared Spectroscopy
*C_f_*	Feed salt concentration (g L^−1^)
*C_f,f_*	Final feed salt concentration (g L^−1^)
CNT	Carbon nanotubes
*C_p_*	Permeate salt concentration (g L^−1^)
DCMD	Direct contact membrane distillation
*d_i_*	Mean inter-fiber space (µm)
*DS_Jw_*	Decay slope of the permeate flux (kg m^−2^ h^−2^)
DTGS–KBr	Deuterated triglycine sulfate–potassium bromide
*E_b_*	Elongation at break (%)
ED	Electrodialysis
FA	Fluorinated acrylate copolymer
FO	Forward osmosis
IPA	Isopropyl alcohol
*J_w_*	Permeate flux (kg m^−2^ h^−1^)
*J_w, average_*	Average permeate flux (kg m^−2^ h^−1^)
*LEP_w_*	Liquid entry pressure of water (Pa)
MD	Membrane distillation
MF	Microfiltration
*M_n_*	Number average molecular weight (kg mol^−1^)
*M_w_*	Mass average molecular weight (kg mol-^1^)
NaCl	Sodium chloride
NaOCl	Sodium hypochlorite
NF	Nanofiltration
PA	Polyamide
PAN	Polyacrylonitrile
PET	Polyester
PP	Polypropylene
PS	Polystyrene
PSf	Polysulfone
PVDF	Polyvinylidene fluoride
PVDF-HFP	Poly(vinylidene fluoride-co-hexafluoropropylene)
RO	Reverse osmosis
SBS	Styrene-butadiene-styrene
SEM	Scanning electron microscope
SGMD	Sweeping gas membrane distillation
SiF	Silica fibers
SiO_2_NPs	Silica nanoparticles
TBAC	Tetrabutylammonium chloride
TFC	Thin film composite
*T_f_*	Feed temperature
*T_p_*	Permeate temperature
*Ts*	Tensile strength (MPa)
UF	Ultrafiltration
VMD	Vacuum membrane distillation
*Y_M_*	Young´s modulus (MPa)
Greek letters:	
α	Rejection factor
*δ*	Thickness (µm)
*δ_PVDF_*	Thickness of the PVDF layer (µm)
*δ_S_*	Thickness of the support layer (µm)
*δ_t_*	Total thickness (µm)
*ε*	Void volume fraction (%)
*θ_w_*	Water contact angle (°)
(*θ_w_*)*_PVDF_*	Thickness of the PVDF layer (°)
(*θ_w_*)*_s_*	Water contact angle of the support layer (°)
Δ*m*	Mass difference (kg)
Δ*t*	Time difference (h)
Δ*T*	Temperature difference between the feed and permeate (K)
